# Comparing Caregiver Perceptions of a Social Robot and Tablet for Serious Game Delivery in Dementia Care: Cross-Sectional Comparison Study

**DOI:** 10.2196/76209

**Published:** 2025-10-07

**Authors:** Dorothy Bai, Kelvin Tan Cheng Kian, Po-Yin Chen, Yeh-Liang Hsu, Gong-Hong Lin

**Affiliations:** 1 School of Gerontology and Long-Term Care, College of Nursing, Taipei Medical University, Taipei, Taiwan; 2 Minor in Applied Ageing Studies programme, S R Nathan School of Human Development, Singapore University of Social Sciences, Singapore; 3 Department of Physical Therapy and Assistive Technology, National Yang Ming Chiao Tung University, Taipei, Taiwan; 4 Gerontechnology Research Center, Yuan Ze University, Taoyuan, Taiwan; 5 International PhD Program in Gerontology and Long-Term Care, College of Nursing, Taipei Medical University, No. 250, Wuxing Street, Xinyi District, Taipei, 110, Taiwan, 886 227361661 ext 3614

**Keywords:** social robot, serious games, dementia, formal caregivers, user experience, usability, technology acceptance

## Abstract

**Background:**

Social robots integrated with serious games hold promise as innovative nonpharmacological strategies in dementia care. However, limited studies have adopted quantitative, platform-level comparisons from the perspective of formal caregivers, who are key stakeholders in technology implementation in dementia care settings.

**Objective:**

This study aimed to evaluate the feasibility, usability, and overall user experience of a serious game–based interaction model delivered via a screen-equipped social robot, compared to a tablet-based version of the same model, from the perspective of formal dementia caregivers.

**Methods:**

A cross-sectional comparative study was conducted with 120 formal dementia caregivers. Each caregiver individually interacted with both a screen-equipped social robot and a touchscreen tablet, delivering identical serious game content incorporating cognitive exercises, music therapy, and reminiscence. The robot featured multimodal interaction capabilities, including voice, gestures, movement, and facial expression display, while the tablet relied on standard touchscreen functions. Caregivers evaluated both platforms using the User Experience Questionnaire (UEQ), System Usability Scale (SUS), and a customized Technology Acceptance Model (TAM). Group comparisons were performed using *t* tests, with post hoc Benjamini-Hochberg correction applied to control for multiple comparisons.

**Results:**

Caregivers generally favored the social robot over the tablet. The robot received higher total UEQ scores (mean 1.29, SD 1.14, vs mean 0.99, SD 1.08; *P*=.004), particularly in enjoyment (*P*=.002), friendliness (*P*=.006), clarity (*P*=.002), organization (*P*=.02), interest (*P*=.01), and innovation (*P*=.002). In the SUS, caregivers rated the robot higher for quick learning (mean 2.71, SD 0.79 vs mean 2.44, SD 0.81; *P*=.002), while overall SUS scores were comparable. TAM results indicated higher total scores for the robot (mean 4.03, SD 0.47 vs mean 3.67, SD 0.58; *P*=.002), with stronger ratings in perceived usefulness (*P*=.002), ease of use (*P*=.002), attitudes (*P*=.002), and behavioral intentions (*P*=.002). All *P* values are from 2-tailed *t* tests and were adjusted using the Benjamini–Hochberg procedure.

**Conclusions:**

The social robot used in this study was perceived by formal dementia caregivers as providing a more favorable user experience and eliciting a stronger intention to use compared to a tablet-based platform. These findings support the feasibility of social robots as a platform for delivering technology-supported activities in dementia care and provide a foundation for future research on their implementation and outcomes in dementia care.

## Introduction

Nonpharmacological interventions have emerged as essential complementary treatments in dementia care, addressing the limitations and side effects associated with pharmacological options [1-3]. Traditional nonpharmacological approaches, however, often encounter practical constraints such as environmental limitations, low engagement from individuals with dementia, and significant reliance on caregiver involvement, increasing caregiver burden and reducing sustainability [3,4]. Advances in smart technologies, including touchscreen devices and software applications, artificial intelligence, and sensor-enabled devices, have enabled new approaches to dementia interventions [5-7]. Among these, serious games offer structured digital activities that aim to enhance cognitive functioning and emotional well-being [8].

Serious games embedded within technological platforms, such as tablets and social robots, hold considerable potential for dementia care by offering engaging and stimulating activities that promote cognitive engagement and social interaction [9]. Social robots, in particular, have shown promise due to their capacity to provide companionship, cognitive stimulation, and emotional support, significantly benefiting individuals living with dementia [9-11]. Integrating serious games into social robots can enhance these benefits by delivering structured, engaging, and personalized interventions [12,13].

Formal caregivers play a pivotal role in adopting and implementing these technological interventions. However, caregivers frequently face significant challenges, including high workload, stress, burnout, and limited resources for meaningful engagement activities [14]. Social robots incorporating serious games can mitigate these challenges by reducing caregiver burden, facilitating engaging interactions, and promoting more efficient and effective caregiving practices. Formal caregivers’ attitudes, usability perceptions, and acceptance levels toward these technologies are therefore critical to successfully integrating social robots with serious games into dementia care settings [14,15].

Despite growing interest in technology-enhanced dementia interventions, research directly comparing caregiver experiences using social robots versus traditional tablets for delivering serious games remains limited. Existing studies often examine these technologies independently rather than comparatively, and many are qualitative in nature, resulting in a significant research gap regarding the relative benefits, usability, and acceptance of social robots compared to more conventional technological devices [16,17]. Moreover, limited studies have adopted quantitative, platform-level comparisons from the perspective of formal caregivers, who are key stakeholders in technology implementation in dementia care settings [18]. This highlights the need for structured evaluations to inform real-world decision-making.

To address this gap, the current study aimed to evaluate the feasibility, usability, and overall user experience of a serious game–based interaction model delivered via a social robot. Specifically, we compared caregivers’ experiences using a screen-equipped social robot with those using a tablet-based version of the same model in dementia care settings. The findings are anticipated to provide practical insights for implementing serious games through different technological platforms and inform future developments in technology-enhanced dementia care.

## Methods

### Study Design and Participants

This study used a cross-sectional comparative design. A total of 120 formal caregivers for individuals living with dementia were recruited from various older adult care institutions. Inclusion criteria were (1) age ≥20 years, (2) currently working as a formal caregiver for persons with dementia, (3) ability to communicate in Mandarin or Taiwanese, and (4) willingness to participate voluntarily with informed consent. Caregivers who provided care for individuals with dementia fewer than 2 days per week were excluded.

### Serious Game Delivery Platforms and Interaction Design

**Figure 1.**
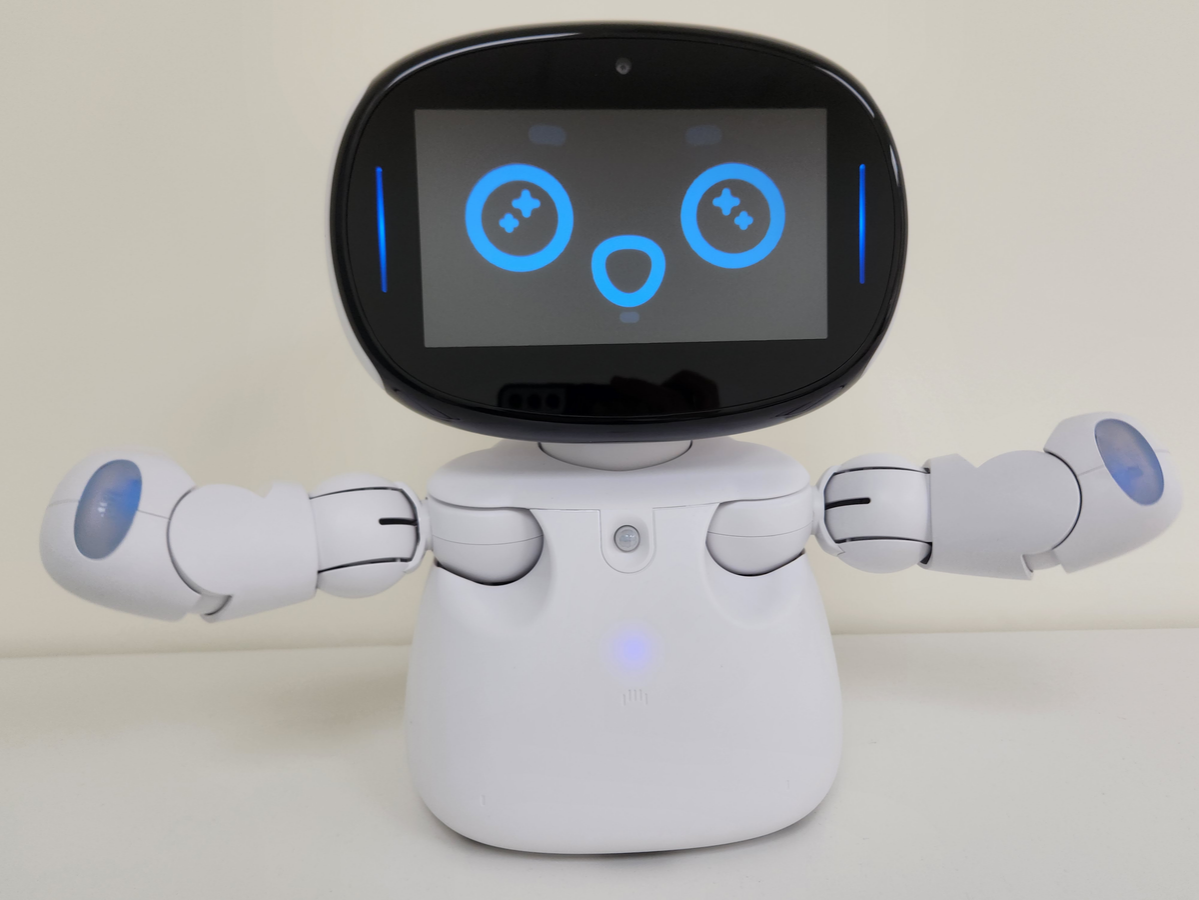
The social robot used in this study to deliver serious games for dementia care.

A screen-equipped social robot with a child-like appearance and multimodal interaction capabilities was selected as the primary platform (Figure 1). The robot is approximately 32 cm tall, weighs 2.5 kg, and includes a screen capable of displaying facial expressions and multimedia content. It features 5 LED lights, 12 degrees of freedom in joint movement, and 5 tactile sensing areas on its head, cheeks, and hands. It is equipped with wireless connectivity, voice recognition, facial and image detection, and an infrared sensor for fall prevention. Mobility is enabled through integrated wheels for dynamic interaction.

The adaptive interaction model embedded in the robot was designed to deliver serious game–based content using established nonpharmacological strategies, including music therapy, reminiscence, and physical activity. While the content was developed primarily for people with dementia, it was intended to be used with caregiver support, especially during initial exposure or setup. The robot was programmed to sing, dance, and perform nostalgic songs to encourage engagement. A suite of cognitive training games was developed to address cognitive domains, including memory, language, spatial awareness, mathematical ability, judgment, abstract thinking, and attention. Each game included 3 difficulty levels and supported both single- and dual-player modes. During the caregiver sessions, both single- and dual-player modes were used. The dual-player mode was available only on the social robot, which featured tactile sensors on its arms and cheeks to detect which participant touched first and thereby determine who could respond to the question.

The robot also supported voice command interaction, allowing users to initiate actions verbally and improving interaction efficiency and engagement. Caregivers explored different difficulty levels based on their interest and available time and were asked to experience all 3 levels in at least one game to familiarize themselves with the difficulty structure. In addition, after each game or question, the robot provided immediate encouraging feedback through facial expressions, movements, and speech to reinforce participation. The same content was implemented on a touchscreen tablet to allow direct comparison of user experience across platforms. Each caregiver interacted with both devices individually for approximately 15‐20 minutes per platform. A summary of the intervention structure and platform-specific features is provided in Table 1.

**Table 1.** Platform-specific features of the social robot and tablet.

Feature	Social robot	Tablet
Primary interaction mode	Touchscreen, voice, gesture, facial expressions	Touchscreen only
Screen size	7-inch LCD touchscreen	10.5-inch touchscreen
Movement or mobility	Yes; uses wheels and joint articulation for movement	None
Additional features	Expressive gestures, motivational verbal feedback, facial recognition	Standard tablet audio/visual feedback
Platform system	Android-based (version 9)	Android-based (version 13)
Content delivered	Cognitive games, music therapy, reminiscence activities	Same as the social robot
Session length per platform	Approximately 15‐20 minutes per platform	Same as the social robot
Game difficulty levels	3 levels, manual selection	Same as the social robot
Player modes	Single-player, dual-player	Single-player
Voice command interaction	Yes; voice commands to initiate actions or respond during games	None
Emotional feedback	Yes; immediate encouraging feedback using expressions, movement, and speech after each action	Limited visual or auditory responses on screen only.

While the serious game content was similar across both platforms, the robot offered additional interaction modalities, including facial expressions, voice commands, physical gestures, and emotional feedback, and also supported a dual-player mode. In contrast, the tablet relied on standard touchscreen input and basic audio-visual feedback. Both the robot and tablet operated on Android-based systems. The software used on both platforms was developed in-house for this study and is not publicly available; however, a demonstration video can be provided upon request for academic or research purposes.

### Data Collection

During this program, only formal caregivers participated; people with dementia were not involved in this evaluation. Caregivers were educated about the social robot and the interaction model and were then given individual, hands-on time to interact with the robot. Afterward, caregivers were educated about the tablet-based version of the same model. We acknowledge that this order may introduce potential order effects, which are discussed further in the limitations section. Caregivers completed all questionnaires based solely on their own user experience with each device. To compare the user experience of the social robot with that of a tablet used in dementia care, the study used 3 widely accepted questionnaires, including the User Experience Questionnaire (UEQ), the System Usability Scale (SUS), and a customized Technology Acceptance Model (TAM) in addition to demographic data such as age, gender, educational level, and duration and frequency of dementia care (Multimedia Appendix 1).

The UEQ, a 12-item tool with a 7-point Likert scale, measures users’ feelings and attitudes toward a product [19]. The SUS, a 10-item questionnaire developed by John Brooke in 1986, assesses usability with responses ranging from “strongly disagree” to “strongly agree,” and converts scores to a 0‐100 scale to gauge system user-friendliness [20]. The TAM, designed by Fred Davis in 1989, evaluates perceived usefulness and ease of use, using a Likert scale to understand users’ beliefs, attitudes, and intentions toward technology adoption [21]. A customized version of the TAM, which includes aspects specific to dementia care to make it relevant to the context of this study, was approved by 3 experts. In this study, TAM items were designed to reflect caregivers’ subjective perceptions, including beliefs about usefulness in dementia care. While not objective clinical assessments, these perceptions are important factors influencing caregivers’ willingness to adopt and use the system. These questionnaires provided a comprehensive assessment of formal caregivers’ experiences with both the social robot and the tablet used in dementia care.

### Data Analysis

Descriptive statistics (mean, SD, frequency, and percentage) were used to summarize demographic and questionnaire data. Group comparisons between the social robot and tablet experiences were conducted using *t* tests for continuous variables. To control for multiple comparisons across UEQ, SUS, and TAM subscale scores, a post hoc Benjamini-Hochberg correction was applied to the resulting *P* values, controlling the false discovery rate at 5% [22]. All analyses were performed using Stata Statistical Software (Release 28; StataCorp LLC) [23], with a significance level set at *P*<.05 and statistical power of 80%.

### Ethical Considerations

This study was approved by the Taipei Medical University Joint Institutional Review Board (approval N202211039) and conducted in accordance with the Declaration of Helsinki and applicable institutional and national guidelines. Written informed consent was obtained from all participants before data collection; participation was voluntary and participants could withdraw at any time without penalty. All collected data were deidentified before analysis and stored on secure, access-controlled servers at Taipei Medical University, with access limited to the study team; hard-copy documents were kept in locked cabinets within secure offices. Data will be retained for 7 years per institutional policy and destroyed thereafter. Participants did not receive compensation for their participation. No identifiable images or personal information are included in this manuscript. No data from care recipients were collected.

## Results

Table 2 presents the demographic and caregiving characteristics of the 120 formal caregivers. The majority were female (103/120, 85.8%) with a mean age of 45.1 (SD 9.3) years. Most participants were care attendants (77/120, 64.2%), followed by nurses (27/120, 22.5%) and other roles (16/120, 13.3%). Educational attainment was mostly at the undergraduate level (85/120, 70.8%), with 11 (9.2%) participants holding postgraduate degrees. Nearly half (53/120, 44.2%) reported having a family member or friend with dementia. In terms of caregiving experience, 50 (41.7%) participants had between 3-<10 years, with an average of 4.3 (SD 1.32) caregiving days per week. The mean perceived care burden score was 5.10 (SD 1.93).

**Table 2.** Demographic data of formal dementia caregivers.

Characteristic	Values
Age (years), mean (SD)	45.1 (12.2)
Sex, n/N (%)	
Male	17/120 (14.2)
Female	103/120 (85.8)
Job position, n/N (%)	
Nurse	27/120 (22.5)
Care attendant	77/120 (64.2)
Other	16/120 (13.3)
Educational level, n/N (%)	
High school and below	24/120 (20.0)
Undergraduate	85/120 (70.8)
Postgraduate	11/120 (9.2)
Any family/friends with dementia, n/N (%)	
Yes	53/120 (44.2)
No	67/120 (55.8)
Years of dementia caregiving, n/N (%)	
Less than 1 year	26/120 (21.7)
1 to less than 3 years	32/120 (26.7)
3 to less than 10 years	50/120 (41.7)
10 years or more	12/120 (10.0)
Weekly care days for dementia (days), mean (SD)	4.30 (1.32)
Care pressure (points), mean (SD)	5.10 (1.93)

As shown in Table 3, caregivers reported significantly higher user experience ratings for the social robot than the tablet across several domains. In the “attractiveness” domain, the robot outperformed the tablet in enjoyment (mean 2, SD 1.43 vs mean 1.43, SD 1.53; *P*=.002) and friendliness (mean 1.43, SD 1.53 vs mean 1.08, SD 1.47; *P*=.006), resulting in a higher total attractiveness score (mean 1.63, SD 1.28 vs mean 1.21, SD 1.78; *P*=.002). In the “perspicuity” domain, the robot was rated as more understandable (mean 1.66, SD 1.51 vs mean 1.28, SD 1.43; *P*=.01) and clearer (mean 1.01, SD 1.61 vs mean 0.64, SD 1.51; *P*=.002), with a higher average perspicuity score (mean 1.06, SD 1.20 vs mean 0.62, SD 1.32; *P*=.002).

**Table 3.** Comparison of User Experience Questionnaire (UEQ) scores between the social robot and tablet among formal dementia caregivers (N=120).

Item	Social robot, mean (SD)	Tablet, mean (SD)	Raw *P* value	BH-adjusted *P* value^a^
Attractiveness				
Enjoyable	2 (1.43)	1.43 (1.53)	<.001	.002
Good	1.42 (1.45)	1.00 (1.56)	.07	.10
Pleasing	0.61 (1.90)	0.68 (1.70)	<.001	.002
Pleasant	0.88 (1.68)	0.67 (1.62)	.04	.05
Attractive	1.15 (1.75)	1.02 (1.61)	.001	.002
Friendly	1.43 (1.53)	1.08 (1.47)	.003	.006
Average score	1.63 (1.28)	1.21 (1.78)	<.001	.002
Perspicuity				
Understandable	1.66 (1.51)	1.28 (1.43)	.007	.01
Easy to learn	1.36 (1.40)	1.22 (1.41)	.22	.27
Easy	0.42 (1.49)	0.86 (1.50)	.008	.01
Clear	1.01 (1.61)	0.64 (1.51)	<.001	.002
Average score	1.06 (1.20)	0.62 (1.32)	.001	.002
Efficiency				
Fast	1.42 (1.53)	1.18 (1.41)	.003	.006
Efficient	1.48 (1.64)	1.25 (1.49)	.56	.63
Practical	0.90 (1.51)	0.44 (1.75)	.95	.98
Organized	1.69 (1.61)	1.19 (1.61)	.02	.02
Average score	0.96 (1.14)	0.97 (1.16)	.99	>.99
Dependability				
Predictable	1.69 (1.58)	0.88 (1.52)	.26	.32
Supportive	1.39 (1.62)	1.09 (1.37)	.06	.09
Secure	1.13 (1.53)	1.08 (1.48)	.75	.81
Meets expectations	1.42 (1.63)	0.93 (1.54)	.19	.24
Average score	1.27 (1.20)	1.12 (1.22)	.15	.20
Stimulation				
Valuable	1.18 (1.49)	0.99 (1.43)	.39	.46
Exciting	1.18 (1.54)	1.09 (1.44)	.01	.02
Interesting	1.05 (1.52)	0.38 (1.78)	.008	.01
Motivating	1.19 (1.57)	1.18 (1.50)	.003	.006
Average score	1.41 (1.32)	1.08 (1.25)	.005	.009
Novelty				
Creative	1.08 (1.46)	0.73 (1.57)	.72	.79
Inventive	1.61 (1.64)	1.09 (1.52)	.02	.03
Leading edge	1.64 (1.52)	1.18 (1.44)	<.001	.002
Innovative	1.61 (1.56)	1.04 (1.46)	<.001	.002
Average score	1.23 (1.34)	0.81 (1.19)	<.001	.002
Average for all items	1.29 (1.14)	0.99 (1.08)	.002	.004

a *P* value adjusted using the Benjamini-Hochberg correction for multiple comparisons.

In terms of “efficiency,” caregivers perceived the robot as faster (mean 1.42, SD 1.53 vs mean 1.18, SD 1.41; *P*=.006) and more organized (mean 1.69, SD 1.61 vs mean 1.19, SD 1.61; *P*=.02). Caregivers also rated the robot higher in “stimulation,” particularly for excitement (mean 1.18, SD 1.54 vs mean 1.09, SD 1.44; *P*=.02) and interest (mean 1.05, SD 1.52 vs mean 0.38, SD 1.78; *P*=.01). In the “novelty” domain, the robot was seen as more inventive (mean 1.61, SD 1.64 vs mean 1.09, SD 1.52; *P*=.03) and cutting-edge (mean 1.64, SD 1.52 vs mean 1.18, SD 1.44; *P*=.002). Overall, the average UEQ score favored the robot (mean 1.29, SD 1.14 vs mean 0.99, SD 1.08; *P*=.004). These differences remained statistically significant after Benjamini-Hochberg correction for multiple comparisons.

As shown in Table 4, caregivers rated the robot significantly higher than the tablet for quick learning (mean 2.71, SD 0.79 vs mean 2.44, SD 0.81; *P*=.002), with statistical significance maintained after Benjamini-Hochberg correction. The total SUS scores were comparable between platforms.

**Table 4.** Comparison of System Usability Scale scores between the social robot and tablet among formal dementia caregivers (N=120).

Item	Social robot, mean (SD)	Tablet, mean (SD)	Raw *P* value	BH-adjusted *P* value^a^
Frequent use likelihood	2.77 (0.83)	2.84 (0.85)	.44	.06
Perceived simplicity	2.22 (0.96)	2.17 (1.01)	.56	.63
Ease of use	2.83 (0.84)	2.74 (0.86)	.39	.46
Independence	2.08 (1.22)	2.06 (1.06)	.80	.86
Function integration	2.69 (0.81)	2.71 (0.76)	.86	.90
Consistency	2.21 (1.01)	2.09 (0.90)	.12	.16
Quick learning	2.71 (0.79)	2.44 (0.81)	.001	.002
Smooth operation	2.50 (0.96)	2.33 (1.01)	.04	.06
Confidence in use	2.90 (0.88)	2.90 (0.77)	>.99	>.99
Minimal learning requirement	2.35 (1.07)	2.22 (1.08)	.10	.13
Total	63.13 (14.01)	61.23 (14.70)	.12	.16

a *P* value adjusted using the Benjamini-Hochberg correction for multiple comparisons.

As summarized in Table 5, caregivers perceived the robot as significantly more useful. It was rated higher for its ability to decelerate cognitive decline (mean 4.15, SD 0.60 vs mean 3.64, SD 0.87; *P*=.002), enhance caregiving effectiveness (mean 3.86, SD 0.70 vs mean 3.49, SD 0.78; *P*=.002), and for overall perceived usefulness (mean 3.99, SD 0.53 vs mean 3.53, SD 0.69; *P*=.002). The robot was also rated higher in perceived ease of use, particularly for interface clarity (mean 4.10, SD 0.60 vs mean 3.84, SD 0.75; *P*=.002) and general ease of use (mean 3.86, SD 0.79 vs mean 3.57, SD 0.81; *P*=.002), resulting in a higher average ease-of-use score (mean 3.95, SD 0.55 vs mean 3.70, SD 0.56; *P*=.002).

**Table 5.** Comparison of caregiver-reported scores on the Technology Acceptance Model between the social robot and tablet (N=120).

Item	Social robot, mean (SD)	Tablet, mean (SD)	Raw *P* value	BH-adjusted *P* value^a^
Perceived usefulness				
Decelerating cognitive decline in people with dementia	4.15 (0.60)	3.64 (0.87)	<.001	.002
Alleviating psychological symptoms for people with dementia	4.03 (0.73)	3.57 (0.81)	<.001	.002
Enhancing social willingness for people with dementia	3.99 (0.76)	3.51 (0.92)	<.001	.002
Enhancing caregiving effectiveness	3.86 (0.70)	3.49 (0.78)	<.001	.002
Improving caregiving performance	3.90 (0.74)	3.41 (0.89)	<.001	.002
Reducing caregiving stress	3.92 (0.78)	3.49 (0.90)	<.001	.002
Improving caregiving simplification	4.06 (0.69)	3.63 (0.83)	<.001	.002
Average score	3.99 (0.53)	3.53 (0.69)	<.001	.002
Perceived ease-of-use				
Interface clarity	4.10 (0.60)	3.84 (0.75)	.001	.002
Sound clarity	4.15 (0.62)	3.88 (0.71)	<.001	.002
Understandability	3.84 (0.83)	3.51 (0.85)	<.001	.002
Overall quality rating	4 (0.69)	3.78 (0.74)	.002	.004
Ease of use	3.86 (0.79)	3.57 (0.81)	.001	.002
Self-resolution of operational issues	3.73 (0.84)	3.58 (0.81)	.06	.08
Ease of becoming familiar and proficient	3.95 (0.72)	3.72 (0.77)	.002	.004
Average score	3.95 (0.55)	3.70 (0.56)	<.001	.002
Attitudes				
User enjoyment	4.29 (0.65)	3.89 (0.77)	<.001	.002
Overall positive view	4.30 (0.67)	3.88 (0.86)	<.001	.002
Average score	4.30 (0.57)	3.89 (0.75)	<.001	.002
Intentions				
Intention to reuse	4.20 (0.71)	3.91 (0.84)	.001	.002
Intention to maintain usage	3.79 (0.87)	3.46 (0.93)	<.001	.002
Intention to recommend to others	4.19 (0.68)	3.82 (0.91)	<.001	.002
Intention to frequently use	4.25 (0.63)	3.83 (0.84)	<.001	.002
Average score	4.11 (0.60)	3.75 (0.74)	<.001	.002
Average for all items	4.03 (0.47)	3.67 (0.58)	<.001	.002

a *P* value adjusted using the Benjamini-Hochberg correction for multiple comparisons.

Attitudinally, caregivers expressed more enjoyment (mean 4.29, SD 0.65 vs mean 3.89, SD 0.77; *P*=.002) and a more favorable overall impression (mean 4.30, SD 0.67 vs mean 3.88, SD 0.86; *P*=.002) of the robot. Intentions to reuse (mean 4.20, SD 0.71 vs mean 3.91, SD 0.84; *P*=.002) and recommend (mean 4.19, SD 0.68 vs mean 3.82, SD 0.91; *P*=.002) were also significantly higher. Statistical significance for these differences was confirmed after Benjamini-Hochberg correction for multiple comparisons.

## Discussion

### Principal Findings

This study evaluated and compared the acceptance and user experience of a screen-equipped social robot and tablet for serious game delivery in dementia care from the perspectives of formal caregivers. While the serious game content was similar across both platforms, the robot incorporated multimodal interaction capabilities, including voice, gestures, movement, and facial expression display, while the tablet relied on standard touchscreen functions. Our findings show that formal caregivers generally favored the robot over the tablet, rating it significantly higher in attractiveness, clarity, stimulation, novelty, quick learning, and perceived usefulness. These findings suggest that social robots may serve as a feasible and engaging option for delivering technology-supported activities in dementia care settings.

### Comparison to Previous Work

Recent studies have shown growing acceptance of social robots among older adults, including those with dementia [9,24-26]. In addition to enhancing the emotional well-being of people with dementia, social robots have also been suggested to help reduce caregivers’ burden by supporting engagement and interaction in care settings [27]. While the perspectives of people with dementia are central, caregivers’ perceptions are also important, as they are key stakeholders in selecting and supporting technologies in dementia care [18]. However, limited studies, particularly quantitative ones, have examined how formal caregivers perceive such tools. Direct comparisons between platforms like social robots and tablets are also rare. By examining caregiver experiences across both platforms, this study offers practical insights for delivering technology-supported activities in dementia care. The following sections discuss our findings in relation to previous work using the UEQ, SUS, and TAM frameworks.

In this study, the social robot outperformed the tablet in most dimensions of the UEQ, particularly in attractiveness, stimulation, and novelty. These findings echo previous research showing that social robots can increase user engagement and motivation among individuals with dementia and their caregivers [14,28,29]. The robot’s physical embodiment, expressiveness, and personalized interaction may contribute to this superior experience, highlighting the importance of multisensory design in serious game delivery for dementia care.

Although total SUS scores between the robot and tablet were similar, the robot was rated significantly higher in “quick learning.” This suggests that while both platforms were usable, the robot provided a more intuitive and seamless experience for caregivers. Such advantages are especially relevant in dementia care, where caregivers benefit from technologies that are easy to learn and integrate into daily routines. These findings are consistent with prior studies reporting generally positive usability ratings for social robots, particularly in contexts requiring minimal training and smooth task execution [16,18].

The significantly higher scores for the social robot across almost all domains of TAM further confirm its perceived utility. According to the Theory of Planned Behavior, perceived behavioral control and positive attitudes are critical to forming behavioral intentions, which influence actual technology adoption [30]. The strong behavioral intentions to reuse and recommend the social robot point toward promising long-term integration into real-world care environments [14]. While caregivers are not positioned to evaluate clinical outcomes, their perceptions, such as whether the system may help slow cognitive decline, are relevant indicators of motivational factors and acceptance in real-world use [31].

### Limitations

This study has several limitations. First, this study evaluated the intervention from the perspective of formal caregivers only; people with dementia were not involved. While caregivers are key stakeholders in technology implementation and offer valuable insights into feasibility and supported use, this limits generalizability to actual end users. Caregivers differ from people with dementia in demographic characteristics, cognitive ability, and technology familiarity, which may affect engagement with the system [14,18,31]. Previous studies showed that people with dementia often respond positively to social robots, though individual preferences and cognitive challenges can affect their engagement [9]. Future research should include direct input from people with dementia to validate usability and inform user-centered design.

Second, this study relied solely on quantitative self-report assessments without behavioral measures or follow-up data, limiting conclusions about real-world usability and adoption. Future studies should include observational or usage data and longitudinal follow-up to assess sustained engagement and outcomes. Third, all participants interacted with the social robot before the tablet, which may have introduced potential order effects. Although this sequence ensured standardized exposure, future studies should consider counterbalanced designs to reduce potential bias. Finally, this study involved multiple statistical comparisons, which may increase the risk of type I error. To address this, a post hoc Benjamini-Hochberg correction was applied, and key results remained significant after adjustment [22].

### Future Directions

While caregivers provide important insights into technology implementation, future research should directly involve people with dementia to assess usability, engagement, and inform user-centered improvements. Studies should incorporate behavioral observations, randomized or counterbalanced designs, and longer-term follow-up to evaluate sustained use and outcomes. Given the social robot’s multimodal and emotionally expressive features, future research should examine how specific elements of robot interaction influence engagement and effects. The refined interaction model may support the design of adaptive tools tailored to diverse cognitive and care needs. Finally, studies should compare platforms such as social robots and tablets in terms of their impact on health outcomes in people with dementia and caregiver burden.

### Conclusions

Serious games offer a promising approach to enhancing nonpharmacological dementia care, and this study demonstrates that, from the perspective of formal caregivers, delivering serious games through a screen-equipped social robot offers a more engaging, user-friendly, and acceptable experience compared with a tablet-based version. The robot’s multimodal interaction capabilities contributed to higher ratings in user experience, perceived usefulness, and behavioral intentions to use. While these results do not allow conclusions about long-term effectiveness or clinical outcomes, they support the perceived feasibility and user acceptance of social robot–mediated serious games from the perspective of dementia caregivers, providing a foundation for future research in this field.

## Supplementary material

10.2196/76209 Multimedia Appendix 1 Survey instruments used in the study, including the User Experience Questionnaire, System Usability Scale, and Technology Acceptance Model.

## Abbreviations

UEQ: User Experience Questionnaire
SUS: System Usability Scale
TAM: Technology Acceptance Model
