# Supplementary material for: Comparing Caregiver Perceptions of a Social Robot and Tablet for Serious Game Delivery in Dementia Care: Cross-Sectional Comparison Study
Source: JMIR Serious Games. 2025 Oct 7;13:e76209. doi: 10.2196/76209 (PMC12505399; doi:10.2196/76209)
Supplement: Multimedia Appendix 1 [file games-v13-e76209-s001.docx]

**User Experience Questionnaire**

|  | **Social Robot** | | | | | | | | | **Tablet** | | | | | | | | |
| --- | --- | --- | --- | --- | --- | --- | --- | --- | --- | --- | --- | --- | --- | --- | --- | --- | --- | --- |
|  |  | **1** | **2** | **3** | **4** | **5** | **6** | **7** |  |  | **1** | **2** | **3** | **4** | **5** | **6** | **7** |  |
| 1 | Annoying | ○ | ○ | ○ | ○ | ○ | ○ | ○ | Enjoyable | Annoying | ○ | ○ | ○ | ○ | ○ | ○ | ○ | Enjoyable |
| 2 | Not understandable | ○ | ○ | ○ | ○ | ○ | ○ | ○ | Understandable | Not understandable | ○ | ○ | ○ | ○ | ○ | ○ | ○ | Understandable |
| 3 | Creative | ○ | ○ | ○ | ○ | ○ | ○ | ○ | Dull | Creative | ○ | ○ | ○ | ○ | ○ | ○ | ○ | Dull |
| 4 | Easy to learn | ○ | ○ | ○ | ○ | ○ | ○ | ○ | Difficult to learn | Easy to learn | ○ | ○ | ○ | ○ | ○ | ○ | ○ | Difficult to learn |
| 5 | Valuable | ○ | ○ | ○ | ○ | ○ | ○ | ○ | Inferior | Valuable | ○ | ○ | ○ | ○ | ○ | ○ | ○ | Inferior |
| 6 | Boring | ○ | ○ | ○ | ○ | ○ | ○ | ○ | Exciting | Boring | ○ | ○ | ○ | ○ | ○ | ○ | ○ | Exciting |
| 7 | Not interesting | ○ | ○ | ○ | ○ | ○ | ○ | ○ | Interesting | Not interesting | ○ | ○ | ○ | ○ | ○ | ○ | ○ | Interesting |
| 8 | Unpredictable | ○ | ○ | ○ | ○ | ○ | ○ | ○ | Predictable | Unpredictable | ○ | ○ | ○ | ○ | ○ | ○ | ○ | Predictable |
| 9 | Fast | ○ | ○ | ○ | ○ | ○ | ○ | ○ | Slow | Fast | ○ | ○ | ○ | ○ | ○ | ○ | ○ | Slow |
| 10 | Inventive | ○ | ○ | ○ | ○ | ○ | ○ | ○ | Conventional | Inventive | ○ | ○ | ○ | ○ | ○ | ○ | ○ | Conventional |
| 11 | Obstructive | ○ | ○ | ○ | ○ | ○ | ○ | ○ | Supportive | Obstructive | ○ | ○ | ○ | ○ | ○ | ○ | ○ | Supportive |
| 12 | Good | ○ | ○ | ○ | ○ | ○ | ○ | ○ | Bad | Good | ○ | ○ | ○ | ○ | ○ | ○ | ○ | Bad |
| 13 | Complicated | ○ | ○ | ○ | ○ | ○ | ○ | ○ | Easy | Complicated | ○ | ○ | ○ | ○ | ○ | ○ | ○ | Easy |
| 14 | Unlikable | ○ | ○ | ○ | ○ | ○ | ○ | ○ | Pleasing | Unlikable | ○ | ○ | ○ | ○ | ○ | ○ | ○ | Pleasing |
| 15 | Usual | ○ | ○ | ○ | ○ | ○ | ○ | ○ | Leading edge | Usual | ○ | ○ | ○ | ○ | ○ | ○ | ○ | Leading edge |
| 16 | Unpleasant | ○ | ○ | ○ | ○ | ○ | ○ | ○ | Pleasant | Unpleasant | ○ | ○ | ○ | ○ | ○ | ○ | ○ | Pleasant |
| 17 | Secure | ○ | ○ | ○ | ○ | ○ | ○ | ○ | Not secure | Secure | ○ | ○ | ○ | ○ | ○ | ○ | ○ | Not secure |
| 18 | Motivating | ○ | ○ | ○ | ○ | ○ | ○ | ○ | Demotivating | Motivating | ○ | ○ | ○ | ○ | ○ | ○ | ○ | Demotivating |
| 19 | Meets expectations | ○ | ○ | ○ | ○ | ○ | ○ | ○ | Does not meet expectations | Meets expectations | ○ | ○ | ○ | ○ | ○ | ○ | ○ | Does not meet expectations |
| 20 | Inefficient | ○ | ○ | ○ | ○ | ○ | ○ | ○ | Efficient | Inefficient | ○ | ○ | ○ | ○ | ○ | ○ | ○ | Efficient |
| 21 | Clear | ○ | ○ | ○ | ○ | ○ | ○ | ○ | Confusing | Clear | ○ | ○ | ○ | ○ | ○ | ○ | ○ | Confusing |
| 22 | Impractical | ○ | ○ | ○ | ○ | ○ | ○ | ○ | Practical | Impractical | ○ | ○ | ○ | ○ | ○ | ○ | ○ | Practical |
| 23 | Organized | ○ | ○ | ○ | ○ | ○ | ○ | ○ | Cluttered | Organized | ○ | ○ | ○ | ○ | ○ | ○ | ○ | Cluttered |
| 24 | Attractive | ○ | ○ | ○ | ○ | ○ | ○ | ○ | Unattractive | Attractive | ○ | ○ | ○ | ○ | ○ | ○ | ○ | Unattractive |
| 25 | Friendly | ○ | ○ | ○ | ○ | ○ | ○ | ○ | Unfriendly | Friendly | ○ | ○ | ○ | ○ | ○ | ○ | ○ | Unfriendly |
| 26 | Conservative | ○ | ○ | ○ | ○ | ○ | ○ | ○ | Innovative | Conservative | ○ | ○ | ○ | ○ | ○ | ○ | ○ | Innovative |

**System Usability Scale**

| **Questions** | **Social Robot** | | | | | **Tablet** | | | | |
| --- | --- | --- | --- | --- | --- | --- | --- | --- | --- | --- |
|  | **Strongly disagree** | **Disagree** | **Neither agree nor disagree** | **Agree** | **Strongly agree** | **Strongly disagree** | **Disagree** | **Neither agree nor disagree** | **Agree** | **Strongly agree** |
| 1. I think I will frequently use this robot / tablet | **1** | **2** | **3** | **4** | **5** | **1** | **2** | **3** | **4** | **5** |
| 1. I think this robot / tablet is too complicated | **1** | **2** | **3** | **4** | **5** | **1** | **2** | **3** | **4** | **5** |
| 1. I think this robot / tablet is simple and easy to use | **1** | **2** | **3** | **4** | **5** | **1** | **2** | **3** | **4** | **5** |
| 1. I think I need someone’s help to use this robot / tablet | **1** | **2** | **3** | **4** | **5** | **1** | **2** | **3** | **4** | **5** |
| 1. I think the functions of this robot / tablet are well integrated. | **1** | **2** | **3** | **4** | **5** | **1** | **2** | **3** | **4** | **5** |
| 1. I think this robot / tablet has many inconsistencies. | **1** | **2** | **3** | **4** | **5** | **1** | **2** | **3** | **4** | **5** |
| 1. I think other people can quickly learn to use this robot / tablet. | **1** | **2** | **3** | **4** | **5** | **1** | **2** | **3** | **4** | **5** |
| 1. I think using this robot / tablet is troublesome. | **1** | **2** | **3** | **4** | **5** | **1** | **2** | **3** | **4** | **5** |
| 1. I feel confident when using this robot / tablet. | **1** | **2** | **3** | **4** | **5** | **1** | **2** | **3** | **4** | **5** |
| 1. I need to learn a lot of information before I can use this robot / tablet. | **1** | **2** | **3** | **4** | **5** | **1** | **2** | **3** | **4** | **5** |

**Technology Acceptance Model**

| **Questions** | **Social Robot** | | | | | **Tablet** | | | | |
| --- | --- | --- | --- | --- | --- | --- | --- | --- | --- | --- |
|  | **Strongly disagree** | **Disagree** | **Neither agree nor disagree** | **Agree** | **Strongly agree** | **Strongly disagree** | **Disagree** | **Neither agree nor disagree** | **Agree** | **Strongly agree** |
| **Perceived usefulness** | | | | | | | | | | |
| 1. I believe this robot / tablet can help slow cognitive decline in the person with dementia. | **1** | **2** | **3** | **4** | **5** | **1** | **2** | **3** | **4** | **5** |
| 1. I believe this robot / tablet can help reduce psychological symptoms like agitation or anxiety. | **1** | **2** | **3** | **4** | **5** | **1** | **2** | **3** | **4** | **5** |
| 1. I believe this robot / tablet encourages the person with dementia to be more socially engaged. | **1** | **2** | **3** | **4** | **5** | **1** | **2** | **3** | **4** | **5** |
| 1. I feel that using this robot / tablet makes my caregiving more effective. | **1** | **2** | **3** | **4** | **5** | **1** | **2** | **3** | **4** | **5** |
| 1. I believe this robot / tablet improves my overall performance as a caregiver. | **1** | **2** | **3** | **4** | **5** | **1** | **2** | **3** | **4** | **5** |
| 1. Using this robot / tablet helps reduce the stress I experience during caregiving. | **1** | **2** | **3** | **4** | **5** | **1** | **2** | **3** | **4** | **5** |
| 1. This robot / tablet makes caregiving tasks easier to manage. | **1** | **2** | **3** | **4** | **5** | **1** | **2** | **3** | **4** | **5** |
| **Perceived ease-of-use** | | | | | | | | | | |
| 1. The interface of this robot / tablet is clear and easy to understand. | **1** | **2** | **3** | **4** | **5** | **1** | **2** | **3** | **4** | **5** |
| 1. The audio or speech from this robot / tablet is clear and easy to hear. | **1** | **2** | **3** | **4** | **5** | **1** | **2** | **3** | **4** | **5** |
| **Questions** | **Social Robot** | | | | | **Tablet** | | | | |
|  | **Strongly disagree** | **Disagree** | **Neither agree nor disagree** | **Agree** | **Strongly agree** | **Strongly disagree** | **Disagree** | **Neither agree nor disagree** | **Agree** | **Strongly agree** |
| 1. It is easy to understand how to use this robot / tablet. | **1** | **2** | **3** | **4** | **5** | **1** | **2** | **3** | **4** | **5** |
| 1. I would rate the overall quality of this robot / tablet as high. | **1** | **2** | **3** | **4** | **5** | **1** | **2** | **3** | **4** | **5** |
| 1. I find this robot / tablet easy to use. | **1** | **2** | **3** | **4** | **5** | **1** | **2** | **3** | **4** | **5** |
| 1. I can usually solve problems on my own when using this robot / tablet. | **1** | **2** | **3** | **4** | **5** | **1** | **2** | **3** | **4** | **5** |
| 1. I became comfortable and proficient with this robot / tablet quickly. | **1** | **2** | **3** | **4** | **5** | **1** | **2** | **3** | **4** | **5** |
| **Attitudes** | | | | | | | | | | |
| 1. I enjoy using this robot / tablet. | **1** | **2** | **3** | **4** | **5** | **1** | **2** | **3** | **4** | **5** |
| 1. I have a positive overall impression of this robot / tablet. | **1** | **2** | **3** | **4** | **5** | **1** | **2** | **3** | **4** | **5** |
| **Intentions** | | | | | | | | | | |
| 1. I intend to use this robot / tablet again in the future. | **1** | **2** | **3** | **4** | **5** | **1** | **2** | **3** | **4** | **5** |
| 1. I would like to continue using this robot / tablet as part of regular care. | **1** | **2** | **3** | **4** | **5** | **1** | **2** | **3** | **4** | **5** |
| 1. I would recommend this robot / tablet to other caregivers. | **1** | **2** | **3** | **4** | **5** | **1** | **2** | **3** | **4** | **5** |
| 1. I would choose to use this robot / tablet frequently if available. | **1** | **2** | **3** | **4** | **5** | **1** | **2** | **3** | **4** | **5** |
